# Abnormal expression of Pygopus 2 correlates with a malignant phenotype in human lung cancer

**DOI:** 10.1186/1471-2407-13-346

**Published:** 2013-07-16

**Authors:** Yang Liu, Qian-Ze Dong, Si Wang, Chang-Qing Fang, Yuan Miao, Liang Wang, Ming-Zhu Li, En-Hua Wang

**Affiliations:** 1 Department of Pathology, The First Affiliated Hospital and College of Basic Medical Sciences of China Medical University, Shenyang 110001, PR China; 2 Department of Medical Microbiology and Parasitology, College of Basic Medical Sciences of China Medical University, Shenyang 110001, PR China

**Keywords:** Pygo2, Lung cancer, Clinicopathological factors, Prognosis, Cell proliferation

## Abstract

**Background:**

Pygopus 2 (Pygo2) is a Pygo family member and an important component of the Wnt signaling transcriptional complex. Despite this data, no clinical studies investigating Pygo2 expression in lung cancer have yet been reported.

**Methods:**

In the present study, the expression patterns of Pygo2 were evaluated by immunochemistry in 168 patients with non-small cell lung cancer (NSCLC). We used small interfering RNA (siRNA) to specifically silence Pygo2, and investigated its effect on cell growth by an 3-(4,5-dimethylthiazol-2-yl)-2,5-diphenyltetrazolium bromide (MTT) assay and flow cytometry analysis in human lung cancer cell lines.

**Results:**

Immunohistochemical analysis showed low expression of Pygo2 in normal lung tissues and increased nuclear expression in lung cancer tissues, either with or without perinuclear expression. Abnormal Pygo2 expression was associated with poor differentiation and a high Tumor (T), Node (N) and Metastases (M) stage in NSCLC patients, and correlated with poor prognosis. Using MTT assay we observed that Pygo2 downregulation inhibited cell proliferation; in addition, flow cytometry analysis showed that Pygo2 knockdown induced apoptosis and increased numbers of G1-phase cells and a reduction in S-phase cells.

**Conclusions:**

We therefore conclude that abnormal Pygo2 protein expression may be a marker for advanced NSCLC. Furthermore, Pygo2 knockdown suppresses cell growth.

## Background

Pygopus proteins are critical elements of the canonical Wnt/β-catenin transcriptional complex which is essential for activation of Wnt target genes [1-4]. In humans, there are two Pygo genes, Pygo1 and Pygo2 [1,5]. Pygo2 has been implicated in Wnt-independent roles in both cancer [6] and development [7,8]. Due to the diverse roles of Pygo2 in cell regulation, disruption of Pygo2 function has been proposed as a strategy for targeting malignant cells [2,9]. In support of this hypothesis, studies have demonstrated that increased Pygo2 expression is specifically required for proliferation of breast and epithelial ovarian cancer cells. Furthermore, silencing of endogenous Pygo2 suppresses the growth of breast and epithelial ovarian cell lines [2,6,10]. Increased expression of nuclear Pygo2 has been reported in 82% of epithelial ovarian cancer tissue samples, compared with tissues from the early stages of the disease [11]. These observations strongly suggest that Pygo2 has an important role in the development of these cancers, although it remains unknown whether Pygo2 has a general role in mammalian cancer development [12-14], and in particular whether Pygo2 is overexpressed in lung cancer tissues. To address this question, we examined Pygo2 expression patterns and their prognostic significance in patients with non-small cell lung carcinoma (NSCLC). Pygo2 protein levels were compared in lung cancer tissues and corresponding normal lung tissues, as well as in A549, SPC-A-1 and LTEP-a-2 lung cancer cell lines. Furthermore, we knocked down Pygo2 with small interfering RNA (siRNA) in human lung cancer cell lines and investigated its effect on cell proliferation.

## Methods

### Patients and specimens

This study was conducted with the approval of the local institutional review board at the China Medical University. Primary tumor specimens were obtained from 168 patients (107 males and 61 females) diagnosed with lung squamous cell carcinoma (SCC) or adenocarcinoma who underwent complete resection at the First Affiliated Hospital of China Medical University between 2002 and 2004. Follow-up information was obtained from a review of the patients’ medical records. None of the patients had received radiotherapy or chemotherapy before surgical resection and all patients were treated with routine chemotherapy after the operation. The mean age of the patients was 60 years (range, 31–87 years). Histological diagnosis and tumor differentiation were determined using hematoxylin and eosin stained tumor tissue sections, according to the World Health Organization (WHO) classification guidelines (2004) [15]. All 168 specimens were re-evaluated with respect to histological subtype, differentiation and tumor stage. Seventy-two cases were classified as SCC and 96 as adenocarcinoma (66 well differentiated, 80 moderately differentiated, and 22 poorly differentiated). Lymph node metastases were identified in 74 of the 168 patients. The TNM (Tumor, Node, Metastasis) staging system of the International Union Against Cancer [16] was used to classify specimens as stage I (*n* = 56), II (*n* = 39), or III (*n* = 73). Of these samples, 30 fresh specimens, including both the tumor tissues and corresponding normal tissues, were also stored at −70°C immediately after resection for protein extraction.

### Cell lines

The immortal human bronchial epithelial cell line, HBE, and the human lung cancer cell lines A549, PG-BE1, NCI-H460, LTEP-a-2 and SPC-A-1 were cultured in either DMEM or RPMI 1640 medium (both from Invitrogen, Carlsbad, CA, USA) supplemented with 10% fetal calf serum (Invitrogen), 100 IU/ml penicillin (Sigma, St. Louis, MO, USA) and 100 μg/ml streptomycin (Sigma). Cells were grown on sterile culture dishes and passaged every 2 days, using 0.25% trypsin (Invitrogen).

The A549 and NCI-H460 cell lines were obtained from the American Type Culture Collection (Manassas, VA, USA). PG-BE1 was kindly gifted by Professor Jie Zheng of Peking University, China. HBE, SPC-A-1 and LTEP-a-2 were obtained from Shanghai Cell Bank (Shanghai, China).

### Immunohistochemistry

Surgically excised tumor specimens were fixed in 10% neutral formalin, embedded in paraffin and then 4-μm-thick sections were prepared. Normal bronchial epithelium present in the tumor tissue samples was used as an internal positive control. Immunostaining was performed using the avidin–biotin–peroxidase complex method (UltrasensitiveTM, MaiXin, Fuzhou, China). Sections were deparaffinized in xylene, rehydrated with graded alcohols, and then autoclaved in 10 μM citrate buffer (pH 6.0) for 2 min. Hydrogen peroxide (0.3% v/v) was applied to block endogenous peroxide activity and sections were incubated with normal goat serum to reduce non-specific antibody binding. Tissue sections were incubated with Pygo2 rabbit monoclonal primary antibodies (1:200 dilution; EPR2024 [2], Abcam, Cambridge, MA, USA). Rabbit immunoglobulin (also at a 1:200 dilution) was used as a negative control. Antibody staining was performed at 4°C overnight. Biotinylated goat anti-rabbit serum immunoglobulin G (IgG) was used as a secondary antibody. After washing, sections were incubated with streptavidin–biotin-conjugated horseradish peroxidase and developed with 3,3′-diaminobenzidine tetrahydrochloride. Counterstaining with hematoxylin was performed and sections were dehydrated in ethanol before mounting. Two investigators independently examined all tumor slides in a randomized manner. Five fields were examined per slide and 100 cells per field were observed at 400× magnification. Evaluation of Pygo2 staining in tissue sections was performed according to the immunohistochemical assessment used by Popadiuk et al. for epithelial ovarian cancer with minor changes [6]. Briefly, the Pygo2 staining intensity was scored as − (no signal), + (weak), ++ (moderate), +++ (strong); (−) to (+) scores were considered to be negative (normal Pygo2 expression) and (++) to (+++) scores were considered to be positive (abnormal Pygo2 expression).

### Western blot analysis

Protein was extracted from tissue samples and cell lines using the Nuclear Protein and Cell Plasma Protein Extraction kit (P0028, Beyotime, Shanghai, China) and protein concentrations were determined with Coomassie brilliant blue (Sigma), using bovine serum albumin (BSA) (Invitrogen) as the standard. Protein samples (50 μg) were separated by 8% or 12% sodium dodecyl sulfate–polyacrylamide gel electrophoresis (SDS-PAGE) and transferred to polyvinylidene fluoride membrane (Millipore, Billerica, MA, USA). After blocking with 1% BSA in Tris-buffered saline (TBS; 20 mM Tris–HCl, 500 mM NaCl) containing 0.05% Tween-20, the membranes were incubated with rabbit anti-Pygo2 monoclonal antibody (1:5000; Abcam) at 4°C overnight. After incubation with horseradish peroxidase-coupled anti-rabbit IgG (SABC, Beijing, China) at 37°C for 2 h, protein bands were visualized using ECL Western Blotting Substrate (Pierce, Rockford, IL, USA) and the BioImaging Systems (UVP Inc., Upland, CA, USA). Protein levels were normalized to β-actin proteins.

### RNA extraction and reverse transcription polymerase chain reaction

Total RNA was extracted from cells using TRIzol Reagent (Invitrogen). Reverse transcription polymerase chain reaction (RT-PCR) was performed using the RNA PCR Kit (AMV) Version 3.0 (TaKaRa Bio Inc., Dalian, Liaoning, China), according to the manufacturer’s instructions. Primer sequences were:

Pygo2-for: 5′-GAAGCGAAGGAAGTCAAATAC-3′;

Pygo2-rev: 5′-GCACAGGACTGCCAAGGAA-3′;

β-actin –for: 5′-AGAGCTACGAGCTGCCTGAC-3′;

β-actin –rev: 5′-AGTACTTGCGCTCAGGAGGA-3′.

After 1.5% agarose gel electrophoresis, the PCR products were visualized using a BioImaging System (UVP Inc.) and quantified using LabWorks Image Acquisition and Analysis Software (UVP Inc.). Pygo2 mRNA levels were normalized to β-actin mRNA.

### Pygo2 siRNA plasmids and transfection

Three different sequences were tested for their ability to downregulate endogenous Pygo2 expression and then used to produce Pygo2 siRNA plasmids (GenePharma, Shanghai, China). The different shDNA sequences were as follows: Sequence A, 5′-GATCCCCGCGAAGGAAGTCAAATACTTTCAAGAGAAGTATT TGACTTCCTTCGCTTTTT-3′ and 5′-AGCTAAAAAGCGAAGGAAGTCAAATA C TTCTCTTGAAAGTATTTGACTTCCTTCGCGGG-3′; Sequence B, 5′-GATCCCCC ACAAGTCCCTTTCCTGGTTTCAAGAGAACCAGGAAAGGGACTTGTGTTTT T-3′ and 5′-AGCTAAAAACACAAGTCCCTTTCCTGGTTCTCTTGAAACCAGGA AAGGGACTTGTGGGG-3′; Sequence C, 5′-GATCCCCGGAGATCCAGTCTGTCT ACTTCAAGAGAGTAGACAGACTGGATCTCCTTTTT-3′ and 5′-AGCTAAAAAG GAGATCCAGTCTGTCACTCTCTTGAAGTAGACAGACTGGATCTCCGGG–3′.

A549, SPC-A-1 and LTEP-a-2 cells were transfected with the Pygo2 siRNA plasmids using Lipofectamine 2000 (Invitrogen), following the manufacturer’s instructions. The empty plasmid was used as a negative control. Transfected cells were tested for Pygo2 expression by Western blotting and RT-PCR.

### Pygo2 cDNA plasmid and transfection

We introduced cDNA plasmids encoding Mus musculus Pygo2 (Gene ID: 68911, GenePharma, Shanghai, China) into Pygo2 knockdown cells, which were transfected with the Homo sapiens Pygo2 siRNA plasmids. The empty plasmid was used as a negative control. Re-expression of Pygo2 was confirmed by Western blot.

### MTT assays

Cells were plated in 96-well tissue culture plates, allowed to attach overnight, and then transfected. Transfected cells were analyzed after 12 h, 24 h, 48 h or 72 h. Cell viability was assessed by the MTT method (Sigma). To each well, 20 μl of 5 mg/ml MTT was added, incubated for 4 h at 37°C, then 200 μl of dimethyl sulfoxide was added to each well and the plates were shaken for 10 min to allow the formazan crystals to dissolve. The optical density at 490 nm was measured using a microplate reader (Model 550, Bio-Rad, USA).

### Flow cytometry

After 48 h, transfected cells harvested from each experimental group were resuspended in 50 μg/ml propidium iodide (Sigma) and incubated for 45 min at room temperature in the dark prior to fluorescence-activated cell sorting analysis. The proportion of cells at each cell cycle phase was determined using a FACS Calibur Flow Cytometer and CellQuest 3.0 software (BD Biosciences, San Jose, CA, USA). Experiments were performed in triplicate.

Annexin V-FITC Apoptosis Kit (BD Pharmingen, USA) was adopted for apoptosis detection according to manufactory’s protocol. Briefly, cells (1 × 10^6^) were transfected with Pygo2 siRNA plasmids and scrambled control, and then collected and resuspended in binding buffer. Annexin V-FITC and propidium iodide were added to each sample and incubated in the dark for 15 minutes before analysis by a FACS Calibur Flow Cytometer. Data were collected and analyzed using CellQuest 3.0 software (BD Biosciences).

### Statistical analysis

All statistical calculations were performed using SPSS 11.5 for Windows software. The χ^2^ test was used to examine possible correlations between Pygo2 expression profiles and clinicopathological factors. The Kaplan–Meier method was used to estimate the probability of patient survival and the log-rank test was used to evaluate differences in survival between patient subgroups. The study paired samples *t*-test was used to compare the data from the densitometry analysis of Western blots for paired normal lung tissues and lung cancer samples. Data from cells in different experimental groups were compared using the independent samples *t*-test. *P* values < 0.05 were considered statistically significant.

## Results

### Nuclear localization of Pygo2 in NSCLC

**Figure 1:**
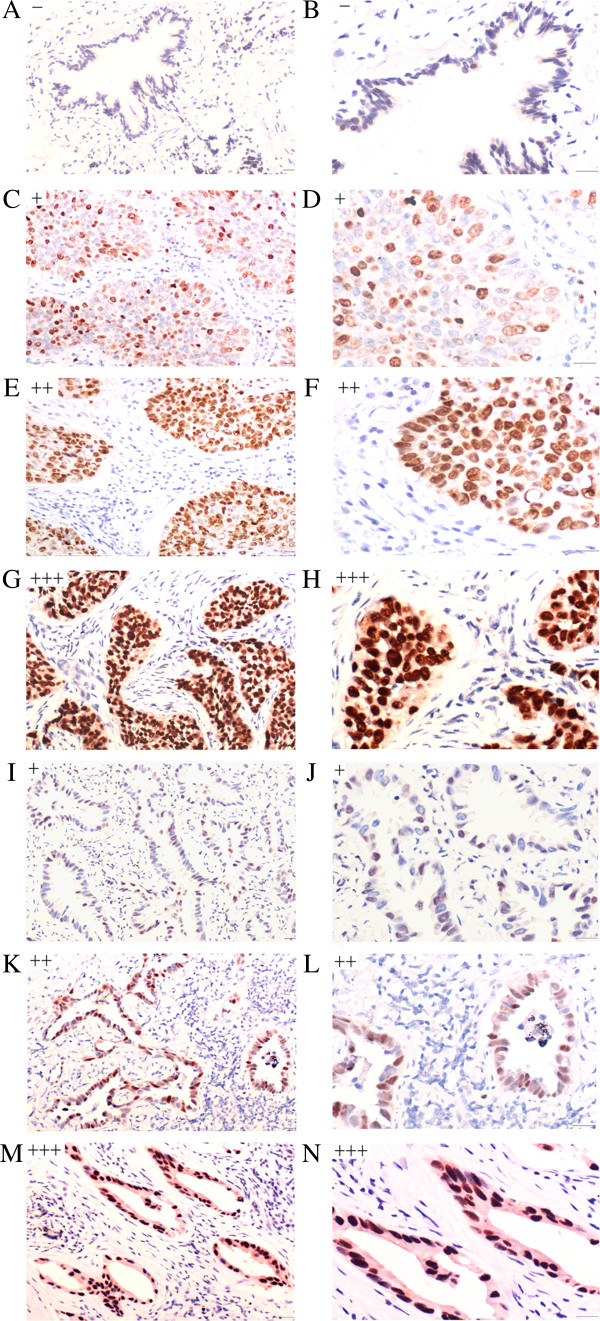
**Pygo2 expression analysis in lung cancer tissues.** Pygo2 staining was classified as negative, weak (light staining; <70% nuclei stained), moderate (intermediate intensity staining; 80% nuclei stained), or strong (intense staining; >90% nuclei stained). Weak or negative Pygo2 staining was detected in the nuclei of normal bronchial epithelium (**A**, **B**). Weak (+) to strong (+++) nuclear accumulation of Pygo2 protein was observed in lung squamous cell carcinoma (**C**-**H**) and adenocarcinoma (**I**-**N**). Bar, 50 μm.

To investigate the abnormalities of Pygo2 expression in NSCLC, we analyzed Pygo2 protein subcellular localization in 168 archived surgical tumor samples. We assessed Pygo2 expression in a semiquantitative manner based on the staining intensity and the percentage of tumor cells with nuclear staining (Figure 1). Pygo2 nuclear expression was undetectable (−) or weak (+) in normal bronchial epithelium. Of the 168 lung cancer samples, 68.45% of NSCLC samples had moderate (++) to strong (+++) nuclear accumulation of Pygo2 protein (see Table 1). These observations suggest a role of nuclear Pygo2 in malignant epithelial cancer.

**Table 1:** Pygo2 expression correlates with specific clinicopathological factors of lung cancer

**Clinicopathological factors**	***n***	**Abnormal expression**	***P *value**
Age			
≤ 60 years	71	50 (70.42%)	0.638
> 60 years	97	65 (67.01%)	
Gender			
Male	107	76 (71.03%)	0.341
Female	61	39 (63.93%)	
Histology			
SCC	72	49 (68.06%)	0.924
Adenocarcinoma	96	66 (68.75%)	
Differentiation			
Well	66	38 (57.58%)	0.015
Moderate–poor	102	77 (75.49%)	
TNM stage			
I–II	95	58 (61.05%)	0.019
III	73	57 (78.08%)	
Lymphatic metastasis			
No	94	59 (62.77%)	0.074
Yes	74	56 (75.68%)	

*SCC* squamous cell carcinoma, *TNM* Tumor, Node, Metastasis.

### Pygo2 overexpression correlates with some NSCLC clinicopathological factors and patient survival

**Figure 2:**
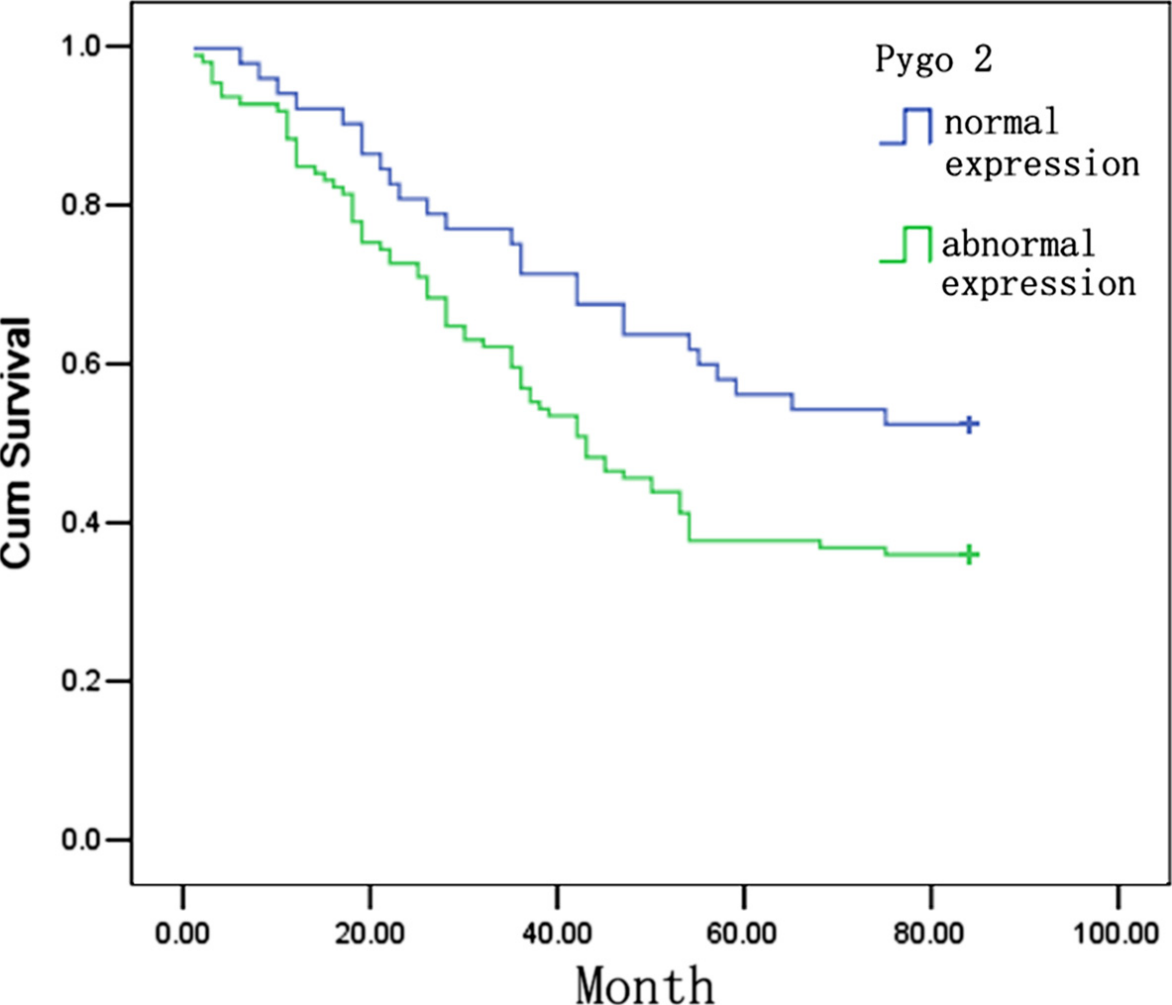
**Pygo2 expression correlates negatively with patient survival.** Kaplan–Meier curves for the analysis of 168 patients with squamous cell carcinoma or adenocarcinoma stratified by Pygo2 expression. Patients with moderate to strong Pygo2 expression had a shorter survival time than patients with normal Pygo2 expression (*P* = 0.030).

We next investigated the associations between abnormal Pygo2 expression and clinicopathological factors. We found that abnormal Pygo2 expression occurred more frequently in advanced tumors (*P =*0.019) with poor differentiation (*P =* 0.015). In contrast, there were no significant correlations between abnormal Pygo2 expression and age, sex, histological type or lymph node metastasis (Table 1). In terms of survival, patients with abnormal Pygo2 expression had a poorer overall survival than patients with normal Pygo2 expression (*P* = 0.030; Figure 2). Overall, our data show that an abnormal Pygo2 expression profile correlates with tumor progression and poor prognosis in NSCLC.

### Increased Pygo2 expression in lung cancer tissues

**Figure 3:**
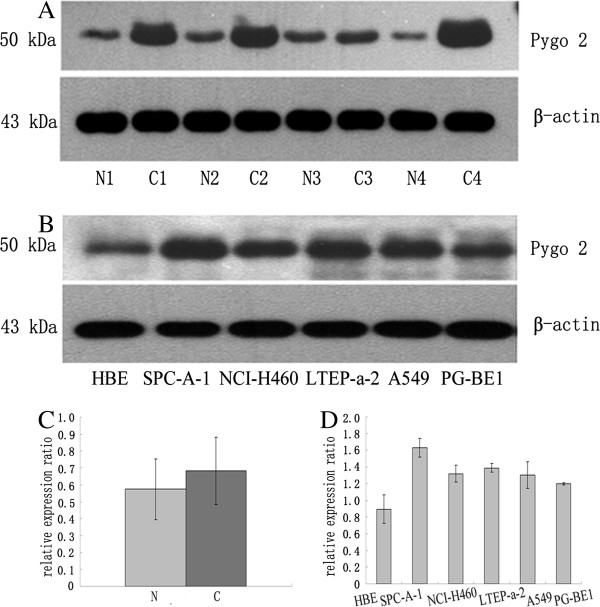
**Pygo2 is overexpressed in lung cancer tissues. **(**A**) Pygo2 (50 kDa band) had increased signal intensity in lung cancer samples (C1–C4) compared with the matched normal lung tissues (N1–N4). (**B**) Increased Pygo2 expression was detected in different lung cancer cells compared to the immortalized bronchial epithelial cell line, HBE. Positions of the molecular weight markers are indicated. Pygo2 protein levels were normalized to β-actin. Statistical analysis showed increased Pygo2 expression in lung cancer tissues (**C**; *P = 0.007*) and cell lines (**D**; SPC-A-1, *P* = 0.038; NCI-H460, *P* = 0.022, LTEP-a-2, *P =0.042*, A549, *P =* 0.047, BE1, *P* = 0.034) compared to matched normal lung tissues.

In normal lung tissues, a major Pygo2 band was detected at 50 kDa. In lung cancer tissues, Pygo2 staining was significantly elevated (*P* = 0.007, *n* = 30; Figure 3A, C). In addition, we examined the levels of Pygo2 expression in normal and lung cancer cell lines (Figure 3B, D). Pygo2 was more highly expressed in lung cancer cell lines than in HBE cells, moreover, the levels of Pygo2 in lung cancer cells were similar to lung cancer tissues, as determined by Western blot analysis. These observations suggest that Pygo2 overexpression is a general characteristic of lung cancer.

### siRNA-mediated Pygo2 silencing

**Figure 4:**
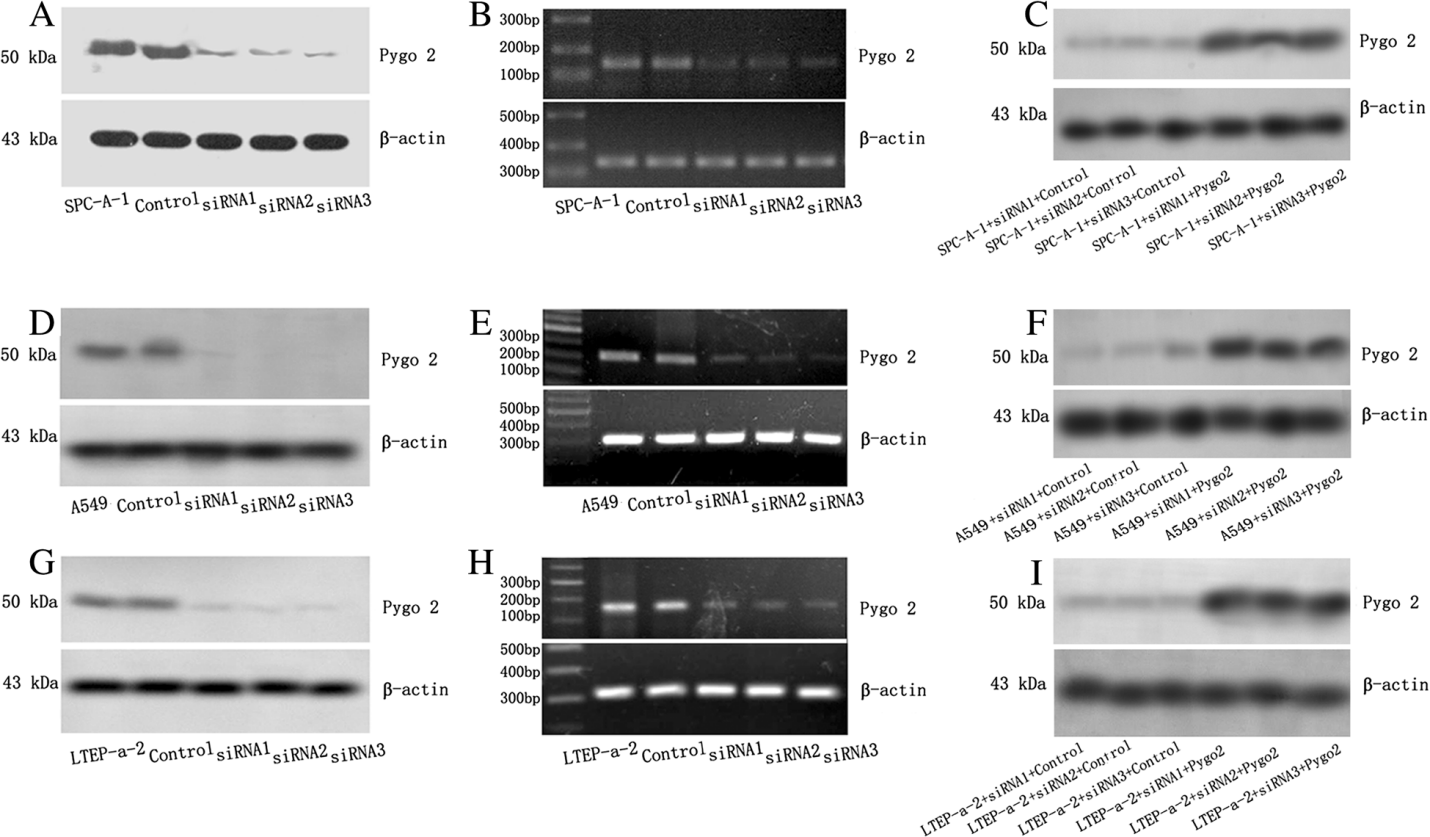
**Pygo2 siRNA reduces Pygo2 expression.** Following transfection with Pygo2 siRNAs, Pygo2 expression was analyzed by Western blotting and RT-PCR. (**A**) Pygo2 protein bands were detected at approximately 50 KDa in untransfected and control SPC-A-1 cells. In contrast, cell lines transfected with sequence C showed reduced Pygo2 expression. (**B**) RT-PCR analysis indicated that Pygo2 mRNA was detected as a specific 170 bp band in untransfected and control SPC-A-1 cells. After transfection with Pygo2 siRNA, expression of Pygo2 mRNA was reduced. (**C**) Lanes 1–3 were siRNA-transfected cells transfected with empty plasmid. Lanes 4–6 were the siRNA-transfected cells transfected with Mus musculus Pygo2 cDNA plasmid. Protein bands representing Pygo2 at approximately 50 kDa were detected. Similar results were observed in A549 (**D**-**F**) and LTEP-a-2 (**G**-**I**) cells.

We used siRNA to downregulate endogenous Pygo2. Of the three sequences tested, one (sequence C) reduced endogenous Pygo2 expression to a level that was barely detectable by Western blotting and RT-PCR. Sequences A and B showed a low efficiency for Pygo2 depletion (data not shown). Following transfection with Pygo2 siRNA sequence C, A549, LTEP-a-2 and SPC-A-1 cells displayed a reduction in endogenous Pygo2 levels. We carried out three independent transfection experiments from each cell line with sequence C and named them Pygo2 siRNA1, siRNA2 and siRNA3. Reduced Pygo2 expression was confirmed by Western blotting and RT-PCR. The empty vector was introduced into each parental cell line and used as a negative control. We also re-introduced Mus musculus Pygo2 cDNA plasmid to confirm that sequence C was specific (Figure 4).

### Pygo2 knockdown inhibits lung cancer cell growth

**Figure 5:**
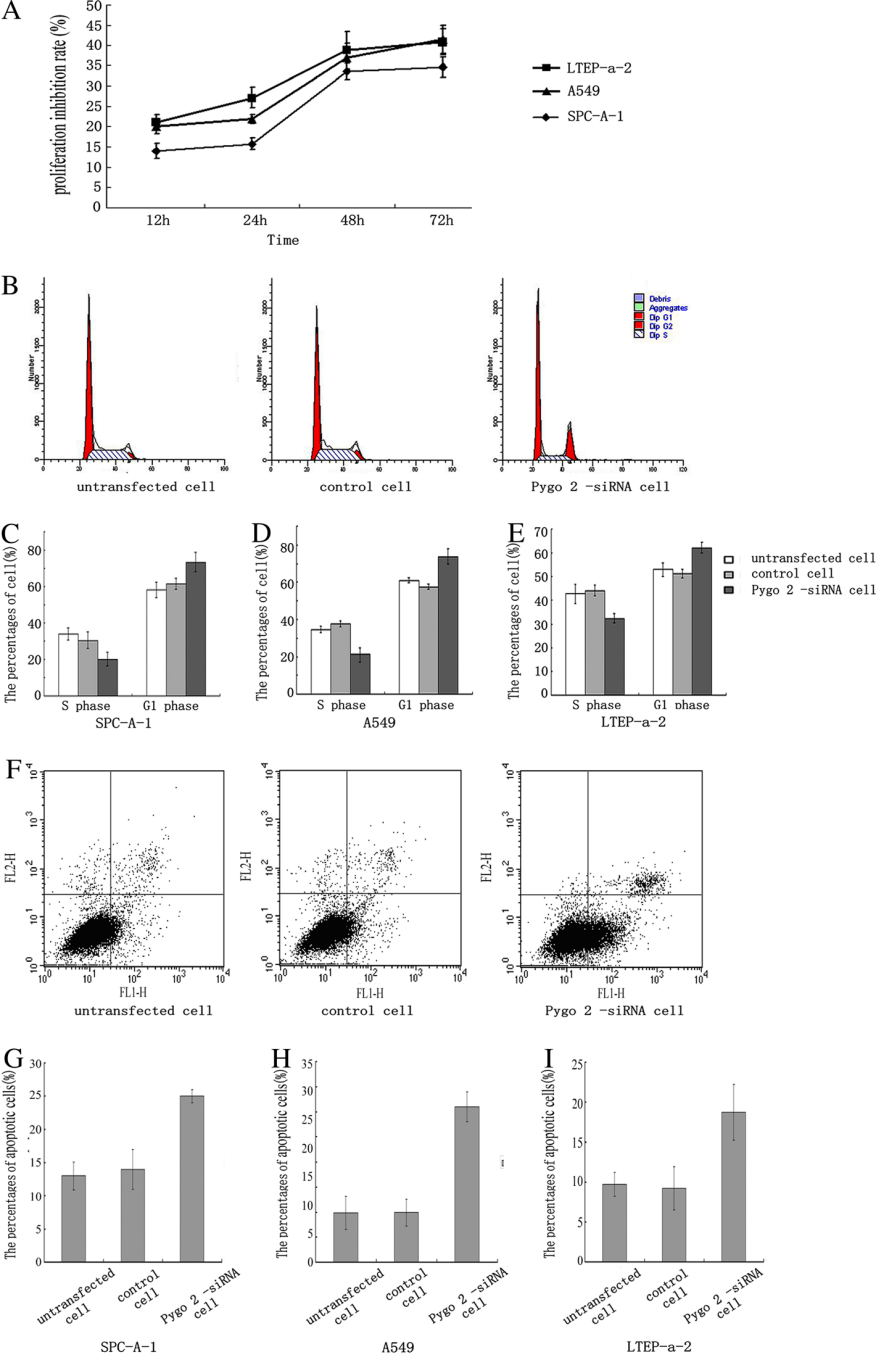
**Pygo2 knockdown inhibits lung cancer cell growth.** (**A**) MTT assays showing that decreased Pygo2 expression inhibited the proliferation of SPC-A-1, A549 and LTEP-a-2 cells. This trend was especially pronounced after 48 h. The y-axis shows the proliferation inhibition rate (%) and the x-axis indicates time after transfection. (**B**) Representative flow cytometry results for SPC-A-1 cell cycle. Treatment with Pygo2 siRNA lead to a significant increase in the proportion of G-phase cells and a significant reduction in S-phase cells, compared with untransfected and control cells. (**C**-**E**) Statistical analysis of the flow cytometry data for SPC-A-1, A549 and LTEP-a-2 cell cycles, respectively. (**F**-**I**) Apoptotic cell death was determined by flow-cytometric analysis with Annexin V and PI staining. The percentage of apoptosis, including early and late stage of apoptotic cell death in each group, is shown. (**F**) Representative results for SPC-A-1 cells showed that knockdown of Pygo2 led to an increase of apoptotic cells compared with untransfected or control cells. (**G**-**I**) Statistical analysis of the flow cytometry data for apoptosis in SPC-A-1, A549 and LTEP-a-2 cell, respectively.

The proliferation rate of siRNA-transfected cells was assessed using the MTT assay (Figure 5A). We observed a time-dependent reduction in Pygo2 expression which was associated with a reduced proliferation rate in all of the lung cancer cell lines, especially at time points later than 48 h. This data shows that Pygo2 silencing significantly inhibited lung cancer cell proliferation.

We next analyzed the cell cycle in transfected cells at the 48 h time point by propidium iodide staining. As shown in Figure 5B–E, Pygo2 silencing resulted in a significant increase in G1-phase cells and a significant reduction in S-phase cells, compared to untransfected cells and negative controls. These data indicated that Pygo2 has the effect on the regulation of the cell cycle of lung cancer cells.

In addition, the Annexin V assay was employed to characterize the apoptotic activities of cells upon Pygo2 knockdown. Clearly, in the cells with Pygo2 knockdown, a significant increase was observed in the population of cells undergoing early and late apoptosis, compared with scrambled controls (Figure 5F–I). These results suggest that Pygo2 knockdown enhances apoptosis of the lung cancer cells.

## Discussion

Abnormal Pygo2 expression in tumor cells has been implicated in tumor progression [2,6,9,10]. However, the pattern of Pygo2 expression in lung cancer was unknown and the correlation of Pygo2 expression with the clinicopathological factors of lung cancer had not been determined. In this study, we demonstrated that Pygo2 protein levels are significantly higher in lung cancer tissues than in normal lung tissues. There was a strong correlation between Pygo2 overexpression and tumor stage. Furthermore, Pygo2 overexpression correlated with poor prognosis in lung cancer patients. In addition, we revealed that siRNA-mediated Pygo2 depletion inhibited the growth of lung cancer cells.

In the present study, we examined the subcellular localization of endogenous Pygo2 in lung tissues by immunohistochemical staining. We demonstrated that Pygo2 protein was weakly detectable or undetectable in the nuclei of normal bronchial epithelia. In contrast, 68.45% of NSCLC samples had moderate to strong nuclear accumulation of Pygo2 proteins. To the best of our knowledge, this is the first report of the Pygo2 expression pattern in NSCLC. Moreover, there was a close correlation between abnormal Pygo2 expression in lung cancer samples and some clinicopathological factors. Abnormal Pygo2 expression was higher in tumors with a greater degree of malignancy (high stage and poor differentiation) and correlated significantly with poor prognosis. These observations are consistent with previous studies indicating that Pygo functions in the nucleus [2,14]. Consistent with our immunohistochemical data, Western blotting showed that Pygo2 protein levels were increased in cancerous tissues compared with normal lung tissues. In addition, we revealed significant overexpression of Pygo2 in lung cancer cell lines relative to immortal bronchial epithelial cells, which further supports our hypothesis that Pygo2 overexpression characterizes lung cancer malignancy.

A previous study also found that Pygo suppression results in cancer cell growth inhibition [6,10]. We therefore reasoned that Pygo2 may regulate the proliferation of lung cancer cell lines. We examined the proliferation rate of A549, LTEP-a-2 and SPC-A-1 cells after siRNA-mediated Pygo2 silencing, and showed that Pygo2 knockdown inhibited cell proliferation, by participating in the regulation of the cell cycle and apoptosis. Therefore, it is probable that Pygo2 overexpression promotes malignant lung cell growth, which may explain our findings that Pygo2 overexpression was the major defining characteristic of NSCLC tumors and correlated with several clinicopathological factors.

How might Pygo2 influence the lung cancer cell growth? A previous study reported that loss of Pygo2 was accompanied by decreased cyclin D1 and c-Myc expression, as well as increased expression of the cell cycle inhibitor p21 [17]. Thus, Pygo2 expression is likely to be finely regulated throughout the cell cycle. These reports suggest that Pygo2 overexpression directly or indirectly regulates critical cell cycle genes, thereby promoting G1–S phase transition. However, the identity of the factors that may participate with Pygo2 to regulate cell cycle progression in lung cancer needs to be further explored. In addition, stable expression of Pygo2 in HeLa cells is reported to exert an anti-apoptotic activity by DNA fragmentation, sub-G1 appearance, loss of mitochondrial membrane potential and the activation of caspase-9 and caspase-3. Meanwhile, Pygo2 also effectively blocks activation of the JNK/AP-1 signaling pathway, thus maintaining the anti-apoptotic Bcl-2 protein in an unphosphorylated state and rendering cells resistant to vinblastine-induced apoptosis [18]. Apoptosis is always associated with cell growth and transformation, escaping from apoptosis susceptibility is a method for tumor growth, and failure in the normal apoptosis pathways contributes to tumorgenicity [19]. Our result also showed that Pygo2 is related to apoptosis of lung cancer cells. Pygo2 mediates cancer cell survival and is therefore essential to the malignant tumor phenotype, and also provides a preliminary insight into how Pygo2 overexpression leads to lung cancer. However, the precise mechanisms need to be defined.

## Conclusion

Our findings show that widespread nuclear Pygo2 overexpression in NSCLC correlates with a malignant phenotype and poor patient survival. In addition, Pygo2 contributes to the proliferation of lung cancer cells, and regulates the apoptosis and cell cycle of lung cancer cells, indicating that Pygo2 is required for lung cancer cell growth. We hypothesize that increased Pygo2 protein expression may serve as a marker of advanced NSCLC, although additional work is needed to identify the specific mechanisms of Pygo2-mediated cancer progression.

## Competing interests

We declare that we have no competing interests.

## Authors’ contributions

Carried out the molecular genetic studies, participated in the sequence alignment and drafted the manuscript: YL and QZD. Participated in the design of the study and performed the statistical analysis: SW and YM. Carried out the immunoassays: CQF and MZL. Conceived of the study, and participated in its design and coordination and helped to draft the manuscript: LW and EHW. All authors read and approved the final manuscript.

## Pre-publication history

The pre-publication history for this paper can be accessed here:

http://www.biomedcentral.com/1471-2407/13/346/prepub

## References

[B1] Thompson B Townsley F Rosin-Arbesfeld R Musisi H Bienz M A new nuclear component of the Wnt signalling pathway Nat Cell Biol 2002 4 5 367 373 10.1038/ncb786 11988739

[B2] Townsley FM Cliffe A Bienz M Pygopus and Legless target Armadillo/beta-catenin to the nucleus to enable its transcriptional co-activator function Nat Cell Biol 2004 6 7 626 633 10.1038/ncb1141 15208637

[B3] Belenkaya TY Han C Standley HJ Lin X Houston DW Heasman J pygopus Encodes a nuclear protein essential for wingless/Wnt signaling Development 2002 129 17 4089 4101 12163411 10.1242/dev.129.17.4089

[B4] Kramps T Peter O Brunner E Nellen D Froesch B Chatterjee S Murone M Zullig S Basler K Wnt/wingless signaling requires BCL9/legless-mediated recruitment of pygopus to the nuclear beta-catenin-TCF complex Cell 2002 109 1 47 60 10.1016/S0092-8674(02)00679-7 11955446

[B5] Li B Mackay DR Ma J Dai X Cloning and developmental expression of mouse pygopus 2, a putative Wnt signaling component Genomics 2004 84 2 398 405 10.1016/j.ygeno.2004.04.007 15234002 PMC2893388

[B6] Popadiuk CM Xiong J Wells MG Andrews PG Dankwa K Hirasawa K Lake BB Kao KR Antisense suppression of pygopus2 results in growth arrest of epithelial ovarian cancer Clin Cancer Res 2006 12 7 Pt 1 2216 2223 16609037 10.1158/1078-0432.CCR-05-2433

[B7] Lake BB Kao KR Pygopus is required for embryonic brain patterning in Xenopus Dev Biol 2003 261 1 132 148 10.1016/S0012-1606(03)00305-1 12941625

[B8] Song N Schwab KR Patterson LT Yamaguchi T Lin X Potter SS Lang RA pygopus 2 has a crucial, Wnt pathway-independent function in lens induction Development 2007 134 10 1873 1885 10.1242/dev.001495 17428831

[B9] Hoffmans R Stadeli R Basler K Pygopus and legless provide essential transcriptional coactivator functions to armadillo/beta-catenin Curr Biol 2005 15 13 1207 1211 10.1016/j.cub.2005.05.054 16005293

[B10] Andrews PG Lake BB Popadiuk C Kao KR Requirement of Pygopus 2 in breast cancer Int J Oncol 2007 30 2 357 363 17203217

[B11] Katoh M WNT signaling pathway and stem cell signaling network Clin Cancer Res 2007 13 14 4042 4045 10.1158/1078-0432.CCR-06-2316 17634527

[B12] Hassan AH Prochasson P Neely KE Galasinski SC Chandy M Carrozza MJ Workman JL Function and selectivity of bromodomains in anchoring chromatin-modifying complexes to promoter nucleosomes Cell 2002 111 3 369 379 10.1016/S0092-8674(02)01005-X 12419247

[B13] Schwab KR Patterson LT Hartman HA Song N Lang RA Lin X Potter SS Pygo1 and Pygo2 roles in Wnt signaling in mammalian kidney development BMC Biol 2007 5 15 10.1186/1741-7007-5-15 17425782 PMC1858683

[B14] Parker DS Jemison J Cadigan KM Pygopus, a nuclear PHD-finger protein required for Wingless signaling in Drosophila Development 2002 129 11 2565 2576 12015286 10.1242/dev.129.11.2565

[B15] Brambilla E Travis WD Colby TV Corrin B Shimosato Y The new World Health Organization classification of lung tumours Eur Respir J 2001 18 6 1059 1068 10.1183/09031936.01.00275301 11829087

[B16] Watanabe Y TNM classification for lung cancer Ann Thorac Cardiovasc Surg 2003 9 6 343 350 15003094

[B17] Gu B Sun P Yuan Y Moraes RC Li A Teng A Agrawal A Rheaume C Bilanchone V Veltmaat JM Pygo2 expands mammary progenitor cells by facilitating histone H3 K4 methylation J Cell Biol 2009 185 5 811 826 10.1083/jcb.200810133 19487454 PMC2711593

[B18] De D Chen A Wu Z Lv S He G Qi Y Overexpression of Pygopus2 protects HeLa cells from vinblastine-induced apoptosis Biol Chem 2009 390 2 157 165 19040349 10.1515/BC.2009.014

[B19] Kitada S Pedersen IM Schimmer AD Reed JC Dysregulation of apoptosis genes in hematopoietic malignancies Oncogene 2002 21 21 3459 3474 10.1038/sj.onc.1205327 12032782

